# Association of physical fitness and motor ability at young age with locomotive syndrome risk in middle-aged and older men: J-Fit^+^ Study

**DOI:** 10.1186/s12877-021-02047-7

**Published:** 2021-01-30

**Authors:** Shaoshuai Shen, Koya Suzuki, Yoshimitsu Kohmura, Noriyuki Fuku, Yuki Someya, Hisashi Naito

**Affiliations:** 1 Institute of Health and Sports Science & Medicine, Juntendo University, 1-1 Hiraka-gakuendai, Inzai, Chiba, 270-1695 Japan; 2 Graduate School of Health and Sports Science, Juntendo University, 1-1 Hiraka-gakuendai, Inzai, Chiba, 270-1695 Japan; 3 Sportology Center, Graduate School of Medicine, Juntendo University, 2-1-1 Hongo, Bunkyo-ku, Tokyo, 113-8421 Japan

**Keywords:** Physical fitness, Motor ability, Locomotive syndrome, Japanese men, Historical cohort, Agility, Middle-aged and older men

## Abstract

**Background:**

Physical fitness and motor ability are associated with the incidence of locomotive syndrome (LS) in older adults. The relationships between physical fitness and motor ability at a young age to LS risk in later life remain unclear. This study examined the association between physical fitness and motor ability among university students and their risk of LS in middle and old age.

**Methods:**

The participants were 231 male alumni aged 48–65 years from the Department of Physical Education of a university in Japan. Physical fitness and motor ability test results during their fourth year at the university were used. Physical fitness tests included the side-step test, vertical jump test, back muscle, grip strength, trunk lift, standing trunk flexion, and step-test. Motor ability was tested using the 50-m and 1500-m run, running long jump, hand-ball throw, and pull-up test. LS risk was assessed using a seven-question standardized self-administered Loco-check questionnaire. Participants were divided into three groups (low, medium, and high) based on physical fitness and motor ability test results at young age, and LS risk was assessed at an older age across the three groups using Cox proportional hazards models.

**Results:**

From the 2017 follow-up survey, the median follow-up period was 37 years (interquartile range, 33–41), and LS risk was suspected for 31 (13.4%) participants. Better performance on the side-step test was associated with the reduced risk of LS (hazard ratio 0.32; 95% confidence interval, 0.101–0.983, *P* = 0.047).

**Conclusions:**

Good agility (side-step test) at a young age may reduce the future risk of LS among middle-aged and older men.

**Supplementary Information:**

The online version contains supplementary material available at 10.1186/s12877-021-02047-7.

## Background

Locomotive syndrome (LS), a condition proposed by the Japanese Orthopaedic Association in 2007, is observed in high-risk individuals with musculoskeletal disease that will likely require nursing care at some point [1, 2]. The number of individuals who are at risk of developing LS after the age of 40 years in Japan is predicted to be 47 million [3]. However, LS does not solely affect middle-aged and older adults whose incidence of LS is approximately 21.1 and 49.3%, respectively; the incidence of LS among men under the age of 40 is approximately 13% [4]. In addition, about 22.3% of men need nursing care due to LS related fractures, falls, and/or musculoskeletal disorders, according to the 2016 Comprehensive Survey of Living Conditions conducted by the Ministry of Health, Labour and Welfare in Japan [5]. Therefore, to prevent nursing care needs of older adults in the future, prevention of LS is necessary from a young age.

LS has been recognized as an important risk factor for falls [6] and reduced mobility in performing activities of daily living (ADL) [4, 7]. Moreover, LS has also been reported to be associated with increased nursing care in older adults in the future [8]; thus, it is necessary to reduce the occurrence of LS. Recently, research on the relationship between physical fitness in older adults and LS has been actively conducted to prevent LS. There have been many study findings indicating that in older adults the risk of LS is associated with static balance [9] and with the back-and-forth postural sway in the balance test [10], timed-up-and-go test [11], walking ability [9], mobility [4], grip strength [12], and back muscle strength [13]. However, it has not been clarified what kind of physical fitness and motor ability at a young age is associated with LS, and whether increasing physical fitness and motor ability at a young age leads to prevention of LS in older adults.

We hypothesized that good physical fitness and motor ability at a young age might reduce the risk of LS in older age. Thus, clarifying the influence of physical fitness and motor abilities in younger individuals on the risk of LS in older age may contribute to early LS risk prevention. To the best of our knowledge this is the first study to investigate the association between physical fitness, motor ability at a young age in a cohort of fourth year university students and the risk of LS in older age Japanese men who graduated from the same university in Japan.

## Methods

### Study design and population

This study is a historical cohort study that included male alumni who graduated from the Department of Physical Education at a university in Japan. The anthropometric, physical fitness tests and motor ability tests are implemented once per year at the university over the 4 years of university studies. The J-Fit^+^ Study is a project that uses the accumulated 50 years of data collected, as indicated above, for research into the association between physical fitness, motor ability at a young age, and future diseases, such as diabetes, type 2 diabetes, and hypertension [14–17]. In the present study, we used data obtained from test results of subjects in their fourth year at the university. All questionnaires and measures in this study do not require a license in order to administer them.

**Fig. 1:**
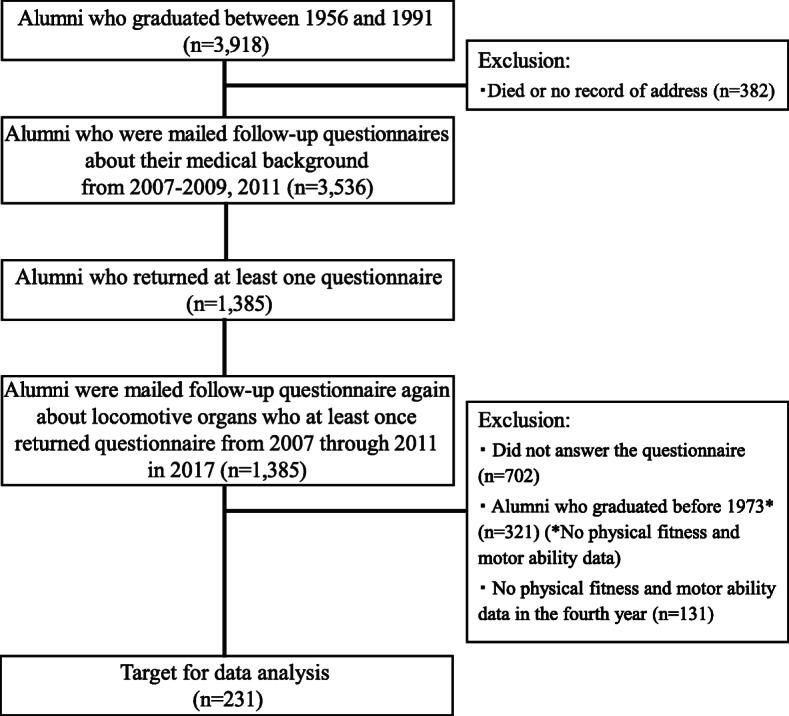
A flow-chart of the participants associated with the present study

There was a total of 3918 male alumni who graduated between 1956 and 1991 who were eligible for the present study. This study did not include female alumni because the Department of Physical Education of the university did not enroll female students until 1991. After excluding 382 participants who had died or had an unknown address, a total of 3536 participants were sent the self-administered questionnaire about their medical background from 2007 to 2009 and in 2011. Of the 3536 participants, 1385 alumni completed and returned questionnaire at least once [16]. In March 2017, another follow-up survey was conducted, involving these 1385 alumni. The alumni received a self-administered questionnaire to obtain information on age, height, weight, body mass index (BMI), daily step counts, and locomotive organs (Loco-check). Individuals (*n* = 702) who did not return the questionnaire were excluded. Furthermore, individuals who graduated before 1973 were also excluded (*n* = 321, because before 1973, our university did not perform physical fitness tests and motor ability tests, and these data were unavailable). In addition, individuals who had no information on physical fitness and motor ability in the fourth year of university were excluded (*n* = 131). Finally, 231 individuals aged 48–65 years were eligible for the analysis. The selection of participants for this study is shown in Fig. 1.

### Physical fitness and motor ability tests

The physical fitness tests consisted of the following seven tests: agility was measured using the side-step test; power was measured using the vertical jump test; muscle strength was measured by back muscle strength and grip strength; flexibility was measured using trunk lift and standing trunk flexion; and endurance was measured using the step-test. The motor ability tests consisted of the following five tests: 50-m run, 1500-m run, running long jump, hand-ball throw, and pull-up. Physical fitness tests and motor ability tests were performed [see Additional file 1] as described in a previous study [18, 19]. In addition, to calculate a comprehensive score that reflected the physical fitness and motor ability levels, we converted scores according to a scoring table for each test [19], as shown in Additional file 2 (Supplementary Table 1 and 2). These comprehensive scores of the physical fitness and motor ability levels were used as the two parameters for analysis. We recorded the physical fitness score as the sum of the side-step, vertical jump, back muscle strength, grip strength, trunk lift, standing trunk flexion, and step-test scores. The motor ability score was recorded as the sum of the 50-m run, 1500-m run, running long jump, hand-ball throw, and pull-up scores.

### Locomotive syndrome risk test

Seven questions were prepared for participants in the Loco-check questionnaire by the Japanese Orthopaedic Association to evaluate locomotive organs [20]. This questionnaire is simple and easy to understand even for older adults [21]. Detailed contents for investigation consisted of the following questions: “1. You cannot put on your sock standing on one leg,” “2. You often trip or slip around the house,” “3. You need to hold on to the handrail when climbing the stairs,” “4. You have difficulty doing moderately heavy housework,” “5. You have difficulty carrying home 2 kg of shopping (e.g., equivalent to two 1-L cartons of milk)”, “6. You cannot walk for a quarter of an hour nonstop,” and “7. You cannot make it across the road before the light turns red.” Subjects answered the Loco-check questions with either “agree” or “disagree.” As described in previous reports [22–24], if the subjects answered “agree” to one or more items on the Loco-check, they were defined as subjects suspected to be at risk of LS (hereafter referred to as the LS-risk group). If they answered “disagree” to all seven items, they were defined as no LS-risk subjects (hereafter referred to as the NLS-risk group).

### Statistical analysis

First, we compared differences in participants’ characteristics between the NLS group and LS-risk group, using the independent samples t-test for continuous variables and the chi-square test for categorical variables.

Next, to identify important factors related to LS risk, physical fitness and motor ability, variables were compared between the NLS and LS-risk groups using the independent samples t-test, and analysis of covariance (ANCOVA). ANCOVA was adjusted for age at the follow-up survey, which is a known factor associated with LS [10, 25]. To identify potential factors correlated with LS risk among the physical fitness variables and those that showed differences in motor ability (*P* value < 0.20) [10] the ANCOVA analyses were adjusted for age, using the aforementioned variables for the subsequent analysis.

Finally, we divided the participants into tertiles (low, medium, and high) based on physical fitness and motor ability variables at university age and compared LS risk across the three groups using Cox proportional hazards models. We obtained both crude and adjusted hazard ratios (HR) with their 95% confidence intervals (CI) for the risk of LS. This analysis was adjusted for the following factors: age, BMI, and daily step counts at the time of the follow-up survey. All analyses were conducted using SPSS Statistics for Windows, Version 21.0 (SPSS Inc., Chicago, IL, USA). A *P* value < 0.05 was considered significant.

## Results

Table 1 shows the basal characteristics of participants in the NLS and LS-risk groups. The median age of the participants at the follow-up survey was 58 years (interquartile range, 54–62). The median follow-up period was 37 years (interquartile range, 33–41). During the follow-up period from May 1973 through to March 2017, LS risk was suspected in 31 (13.4%) participants. Among participants in the LS-risk group, weight and BMI at the follow-up survey were significantly higher than in the participants in the NLS group (*P* < 0.05).

**Table 1:** Characteristics of participants

	All (*n* = 231)	NLS risk group (*n* = 200)	LS-risk group (*n* = 31)	*P*-value^a^
Characteristics at follow-up survey				
Age, years	58.0 (54.0, 62.0)	58.0 (54.0, 62.0)	59.0 (53.0, 64.0)	0.332
Height, cm	172.0 (168.0, 177.0)	172.3 (168.5, 177.1)	172.0 (167.0, 176.0)	0.888
Weight, kg	71.0 (65.0, 78.0)	70.5 (65.0, 77.0)	72.0 (68.0, 80.0)	0.048
BMI, kg/m^2^	23.8 (22.1, 25.5)	23.7 (22.0, 25.4)	24.4 (23.3, 26.5)	0.020
Daily step counts, steps/day	5000.0 (0.0, 8000.0)	4000.0 (0.0, 8000.0)	5000.0 (1500.0, 6000.0)	0.939
Smoking status, n (%)				
Never smoker	94 (40.7)	86 (43.0)	8 (25.8)	0.070
Current smoker	37 (16.0)	29 (14.5)	8 (25.8)	0.110
Former smoker	100 (43.3)	85 (42.5)	15 (48.4)	0.538
Drinking status, n (%)				
None	23 (10.0)	22 (11.0)	1 (3.2)	0.329
Current	194 (84.0)	166 (83.0)	28 (90.3)	0.431
Former	14 (6.0)	12 (6.0)	2 (6.5)	1.000
Follow-up period, years	37.0 (33.0, 41.0)	36.5 (33.0, 41.0)	38.0 (32.0, 43.0)	0.355
Characteristics in fourth year at university				
Current working status, yes (%)	193.0 (83.5)	168.0 (84.0)	25.0 (80.6)	0.220
Age, years	21.0 (21.0, 21.0)	21.0 (21.0, 21.0)	21.0 (21.0, 21.0)	0.564
Height, cm	172.8 (168.8, 177.8)	172.8 (168.8, 177.8)	173.3 (168.4, 176.7)	0.864
Weight, kg	65.5 (61.5, 71.0)	65.5 (61.5, 71.0)	66.4 (61.5, 72.5)	0.740
BMI, kg/m^2^	22.0 (21.0, 23.2)	22.0 (21.0, 23.2)	22.2 (21.3, 23.5)	0.801
Year of graduation	1981 (1977, 1985)	1981 (1977, 1985)	1980 (1975, 1986)	

The data are presented as medians (interquartile range) for continuous variables and number (percentage) for categorical variables

*BMI* body mass index, calculated as weight in kilograms divided by height in meters squared, *LS* locomotive syndrome, *NLS risk group* answered “disagree” to all seven items on “Loco-check”; *LS-risk group* “agree” to one or more items on “Loco-check”

^a^ *P*-value of independent-samples *t*-test (for continuous variables) or chi-square test (for categorical variables) between NLS risk group and LS-risk group

Table 2 shows the differences between the NLS group and the LS-risk group in physical fitness tests and motor ability tests. There were no significant differences in all test items.

**Table 2:** Comparison between the NLS group and LS-risk group on physical fitness tests and motor ability tests

	All (*n* = 231)	NLS risk group (*n* = 200)	LS-risk group (*n* = 31)	*t*-test *P*-value^a^	ANCOVA *P-*value^b^
Physical fitness tests					
Side-step test, point	51.19 (4.2)	51.37 (4.3)	50.10 (3.2)	0.114	0.133
Vertical jump test, cm	63.84 (6.6)	63.71 (6.7)	64.74 (6.1)	0.420	0.337
Back muscle strength, kg	171.96 (30.2)	171.69 (30.2)	173.71 (30.5)	0.729	0.520
Grip strength, kg	51.42 (6.3)	51.34 (6.1)	51.97 (7.1)	0.604	0.617
Trunk lift, cm	59.22 (7.2)	59.11 (6.9)	59.97 (9.2)	0.538	0.713
Standing trunk flexion, cm	14.61 (5.6)	14.42 (5.6)	15.90 (5.8)	0.171	0.211
Step-test^c^	73.37 (14.4)	73.83 (14.0)	70.43 (16.4)	0.222	0.250
Physical fitness scores, point	28.13 (2.1)	28.13 (2.1)	28.13 (1.9)	0.992	0.900
Motor ability tests					
50-m run, s	7.07 (0.3)	7.07 (0.3)	7.03 (0.3)	0.534	0.434
1500-m run, s	331.37 (31.8)	331.45 (32.4)	330.90 (28.5)	0.930	0.839
Running long jump, cm	528.99 (43.5)	528.88 (43.4)	529.71 (44.3)	0.921	0.795
Hand-ball throw, m	31.45 (4.1)	31.31 (4.0)	32.32 (4.5)	0.199	0.211
Pull-up, point	14.33 (5.4)	14.37 (5.5)	14.10 (4.7)	0.795	0.826
Motor ability scores, point	63.90 (12.2)	63.74 (12.2)	64.97 (12.4)	0.602	0.505

The data are presented as the mean value (standard deviation)

*NLS* answered “disagree” to all seven items on “Loco-check”, *LS* locomotive syndrome, *LS* “agree” to one or more items on “Loco-check”

ANCOVA: analysis of covariance

^a^ *P* value of independent-samples *t*-test. ^b^Age-adjusted at follow-up survey. ^c^ Step-test scored using the index derived from the formula shown in Additional file 1

Since the side-step test was confirmed as an important factor related to LS risk, a Cox proportional hazards model analysis for the variable was performed (Table 3). In an unadjusted analysis, the side-step test (agility) was not significantly associated with the risk of LS (Model 1). After adjusting for age (continuous variable) at the university (at baseline), the side-step test (agility) was still not significantly associated with the risk of LS (Model 2). As seen in Model 2, it is confirmed that the age at baseline had no effect on the relationship between the side-step test (agility) and the risk of LS. Therefore, in Models 3 and 4, the age at baseline was not included in the analysis as a covariate. However, previous studies have shown that the current age has an impact on the risk of LS [10, 25]. Therefore, in Models 3 and 4, we added the age at the time of the follow-up survey as a covariate to the analysis. In another model, age (continuous variable) and BMI tertiles (low, medium, high) at the follow-up survey were entered as adjusted factors instead of age (continuous variable) at the university (Model 3). In Model 3, the risk of LS was significantly lower in participants with high side-step test results (agility) than in participants with low side-step test (agility) (HR 0.32; 95% CI, 0.102–0.986, *P* = 0.047). In Model 4, which included Model 3 plus the daily step counts (steps/day) at the follow-up survey were entered as adjusted factor. The high side-step test (agility) participants had a significantly lower risk of LS than low side-step test (agility) participants (HR 0.32; 95% CI, 0.101–0.983, *P* = 0.047).

**Table 3:** Hazard ratios of the risk of locomotive syndrome according to Side-step test (agility) fitness level

		LS-risk	Unadjusted model		Adjusted model					
		group	(Model 1)		(Model 2)		(Model 3)		(Model 4)	
	All	n (%)	HR (95% CI)	*P-* value	HR (95% CI)	*P-* value	HR (95% CI)	*P-* value	HR (95% CI)	*P-* value
Side-step test, point (agility)		31 (13.4%)								
Low ≤50	90	18 (20.0%)	1.00 (Reference)		1.00 (Reference)		1.00 (Reference)		1.00 (Reference)	
51 ≤ Medium ≤53	80	9 (11.3%)	0.71 (0.315–1.591)	0.403	0.66 (0.289–1.502)	0.321	0.58 (0.239–1.391)	0.221	0.57 (0.238–1.385)	0.217
High ≥54	61	4 (6.6%)	0.45 (0.151–1.337)	0.150	0.43 (0.142–1.272)	0.126	0.32 (0.102–0.986)	0.047	0.32 (0.101–0.983)	0.047

Model 1 was the unadjusted model

Model 2 included Model 1 plus age (continuous variable) at university (at the baseline) was adjusted as covariates

Model 3 included Model 1 plus age (continuous variable), and body mass index (low, medium, high) at the follow-up survey were entered as adjusted factors

Model 4 included Model 3 plus the daily step counts at the follow-up survey, steps/day, is entered as adjusted factor

*HR* hazard ratios, *CI* confidence interval. The data are presented as the hazard ratio (95% confidence interval [CI])

Tables 4 and 5 show hazard ratios of the risk of LS according to the physical fitness and the motor ability levels. No significant associations with the risk of LS were found.

**Table 4:** Hazard ratios of the risk of locomotive syndrome according to physical fitness level, except the side-step test

		LS-risk	Adjusted model	
		group		
	All	n (%)	HR (95% CI)	*P* value
Physical fitness tests				
Vertical jump test, cm		31 (13.4%)		
Low ≤61	81	8 (9.9%)	1.00 (Reference)	
62 ≤ Medium ≤67	83	13 (15.7%)	2.00 (0.564–7.060)	0.284
High ≥68	67	10 (14.9%)	2.81 (0.733–10.736)	0.132
Back muscle strength, kg		31 (13.4%)		
Low ≤158	77	9 (11.7%)	1.00 (Reference)	
159 ≤ Medium ≤181	79	12 (15.2%)	1.95 (0.609–6.268)	0.260
High ≥182	75	10 (13.3%)	1.23 (0.319–4.763)	0.761
Grip strength, kg		31 (13.4%)		
Low ≤49	86	12 (14.0%)	1.00 (Reference)	
50 ≤ Medium ≤53	68	7 (10.3%)	0.72 (0.207–2.483)	0.599
High ≥54	77	12 (15.6%)	0.83 (0.265–2.621)	0.756
Trunk lift, cm		31 (13.4%)		
Low ≤56	79	12 (15.2%)	1.00 (Reference)	
57 ≤ Medium ≤62	81	6 (7.4%)	0.32 (0.096–1.052)	0.061
High ≥63	71	13 (18.3%)	1.58 (0.484–5.168)	0.449
Standing trunk flexion, cm		31 (13.4%)		
Low ≤12	88	10 (11.4%)	1.00 (Reference)	
13 ≤ Medium ≤17	74	9 (12.2%)	1.13 (0.352–3.632)	0.837
High ≥18	69	12 (17.4%)	1.63 (0.519–5.118)	0.402
Step-test^a^		31 (13.4%)		
Low ≤65	77	13 (16.9%)	1.00 (Reference)	
66 ≤ Medium ≤80	79	10 (12.7%)	0.60 (0.203–1.761)	0.351
High ≥81	75	8 (10.7%)	0.83 (0.216–3.175)	0.784
Physical fitness scores, point		31 (13.4%)		
Low ≤27	85	10 (11.8%)	1.00 (Reference)	
28 ≤ Medium ≤29	87	13 (14.9%)	0.86 (0.273–2.714)	0.798
High ≥30	59	8 (13.6%)	0.62 (0.139–2.767)	0.531

HR, hazard ratio; CI, confidence interval. The data are presented as the hazard ratio (95% confidence interval [CI]). In adjusted model the results of physical fitness tests and motor ability tests (low, medium, high), age (continuous variable), body mass index (low, medium, high) and daily step counts at the follow-up survey were entered as adjusted factors

^a^ Step-test scored using the index derived from the formula shown in the eMaterials

**Table 5:** Hazard ratios for the risk of locomotive syndrome according to motor ability level, except the side-step test

		LS-risk	Adjusted model	
		group		
	All	n (%)	HR (95% CI)	*P* value
Motor ability tests				
50-m run, s		31 (13.4%)		
Low ≤6.9	89	11 (12.4%)	1.00 (Reference)	
7.0 ≤ Medium ≤7.2	77	15 (19.5%)	2.14 (0.780–5.843)	0.140
High ≥7.3	65	5 (7.7%)	0.31 (0.058–1.613)	0.162
1500-m run, s		31 (13.4%)		
Low ≤318	79	13 (16.5%)	1.00 (Reference)	
319 ≤ Medium ≤342	75	9 (12.0%)	0.53 (0.173–1.633)	0.270
High ≥343	77	9 (11.7%)	0.62 (0.142–2.691)	0.521
Running long jump, cm		31 (13.4%)		
Low ≤514	79	11 (13.9%)	1.00 (Reference)	
515 ≤ Medium ≤545	78	11 (14.1%)	0.41 (0.099–1.661)	0.210
High ≥546	74	9 (12.2%)	0.40 (0.091–1.778)	0.229
Hand-ball throw, m		31 (13.4%)		
Low ≤30	94	10 (10.6%)	1.00 (Reference)	
31 ≤ Medium ≤33	73	8 (11.0%)	0.79 (0.228–2.712)	0.704
High ≥34	64	13 (20.3%)	0.54 (0.137–2.094)	0.370
Pull-up, point		31 (13.4%)		
Low ≤11	82	9 (11.0%)	1.00 (Reference)	
12 ≤ Medium ≤17	82	15 (18.3%)	2.01 (0.650–6.191)	0.226
High ≥18	67	7 (10.4%)	1.05 (0.258–4.266)	0.947
Motor ability scores, point		31 (13.4%)		
Low ≤58	80	9 (11.3%)	1.00 (Reference)	
59 ≤ Medium ≤70	80	10 (12.5%)	0.50 (0.102–2.431)	0.388
High ≥71	71	12 (16.9%)	0.84 (0.090–7.787)	0.876

The data are presented as the hazard ratio (95% confidence interval [CI])

In adjusted model, the results of physical fitness tests and motor ability tests (low, medium, high), age (continuous variable), body mass index (low, medium, high) and Daily step counts at the follow-up survey were entered as adjusted factors

*HR* hazard ratios, *CI* confidence interval

## Discussion

This study examined the association of physical fitness and motor ability of university-aged students with the risk of LS in middle-aged and older Japanese men. The results showed that the risk of LS was lower in high side-step test (agility) participants than in low side-step test (agility) participants at a young age. The results of this study demonstrate the good agility at a young age contributes to a lower risk of LS at middle and old age.

During the follow-up period, the risk of LS was suspected for 31 (13.4%) participants in this study. However, Sasaki et al. reported the risk of LS in Japan for 56 (21.2%) men, among the 264 men whose mean ages were 56.3 ± 14.1 (21–86) years [22]. Although the age of the subjects in the previous study is similar to the age of the subjects in this study, and LS test methods consisting of only the Loco-check questionnaire, the proportion of people suspected of having LS risk in our study was lower, probably owing to the greater physical fitness and motor ability of our subjects.

Overall, 231 of the 1385 individuals’ second follow-up survey in 2017 (16.7%) met the criteria for the present study. The median year of graduation of the participants in the present study at the university (at the baseline) was 1981. We thus compared the physical fitness and motor ability test results of the subjects in the present study with male students in their fourth year of study at Juntendo University in 1981 (Supplementary Table 3). Although we had no way to conduct statistical analyses, for all tests, the differences are very small, indicating that the sample of our study can represent the data of alumni who graduated from the Department of Physical Education of a university. Therefore, we believe that the dropout data have little effect on the results of this study. The median age of the participants in the present study at the university (at the baseline) was 21 years; we therefore also compared the results of the physical fitness and motor ability tests of the subjects in the present study to those of age peers in general who were 21 years old in 1981 (Supplementary Table 3). In addition, although we have no way to conduct statistical analysis, for all tests, the results of the subjects in the present study are better than those of age peers in general in 1981, indicating that the subjects of our study are likely to have better physical fitness and motor ability. This reveals that further investigation is needed to determine the effects of physical fitness and motor ability at young age on LS risk and compare them with age peers in general.

Agility has been identified as the ability to include whole-body change of direction as well as change of limb direction [26, 27], the ability to coordinate, quickly and accurately, the big muscles of the body in a particular activity (a neurological function) [28]. This definition suggests that ability involves modulated movements and physical reactions. If agility is a concept of harmony, then it can also be considered as physical control, with muscle control being an integral part of agility. Since the side-step test includes the concepts mentioned above, this test is an accepted way to measure agility, and is also considered an effective indicator. In LS, the three main components of the locomotive system are the bones (support), joints and intervertebral discs (mobility, shock absorption), and the muscular and nervous systems (drive, control) [29]. Therefore LS is identified as a condition in which mobility functions such as sit-to-stand or gait, are reduced as a result of locomotive organ/system impairment [1]. Although agility and LS are essentially two completely different concepts, we can see that agility has many determinants that are common to LS. For example, the whole-body change of direction as well as change of limbs in the definition of agility is actually dependent on the support of bones and the help of joints. In addition, modulation of movements in the definition of agility and the nervous system in LS refer to the individual’s ability to control the body and muscles. Hence agility may be a predictor of the risk of LS.

In this follow-up survey, 15 (6.5%) participants were suspected of having LS risk owing to their inability to “cross the road before the light turns red” (Supplementary Table 4), implying that the subjects are unable to move or walk quickly within a limited time. The side-step test (agility) assesses the number of times the subject can quickly move left and right within a specified time (Additional file 1). This may imply that if one has poor agility when one is young, one’s ability to move quickly within a limited period of time during middle and old age will be poor, leading to LS.

Furthermore, our results support past findings with respect to the association between physical fitness and LS risk in the older adult population. Yoshimura et al. found that slower Five Times Sit-to-Stand Test times (lower extremity strength) were associated with a higher stage of LS in middle-aged and older individuals [4]. Negrete and Brophy reported that the single-leg isokinetic squat strength (lower extremity strength) was associated with complex multi-directional tasks over short distances (agility) in university-aged subjects [30]. In addition, Pembrey et al. concluded in their study that agility, and jumping ability (lower extremity strength) could assess the same physical attributes in young competitive-level team sports players [31]. These authors showed that lower extremity strength played an important role in agility among university-aged subjects. In addition, 12 (5.2%) participants were suspected of having LS risk owing to the need for holding on to the handrail when climbing the stairs (Supplementary Table 4), implying that the strength of the lower limbs of these individuals has decreased. As mentioned previously, the strength of the lower limbs plays a very important role in agility. Thus, maintaining good strength of the lower limbs at a young age ensures that the strength of the lower limbs will not decline quickly in middle and old age. We believe that although the relationship between the lower limb strength of young and older adults is equivocal; both may be positively correlated. Therefore, based on these findings, agility at a young age may be a sensitive factor for predicting the risk of LS, and may indirectly help prevent the progression of LS.

However, this association was not confirmed in Model 1. This is because LS risk includes several important factors, including age, which were not considered in Model 1. Age is considered to be an important factor in LS risk [10, 25]. Nevertheless, agility was still not associated with risk of LS when a young age was considered in Model 2. This is because, among the 231 participants, 205 (88.7%) were aged 21 years, 23 (10.0%) were aged 22 years, and three (1.3%) were aged 23 years at the fourth year at university (at baseline). This result (Model 2) suggested that this difference did not introduce confounding effects. Therefore, in Model 3 we identified a negative relationship in terms of agility and the risk of LS when age and BMI at the follow-up survey were considered. The reasons for considering BMI in Model 3 is that BMIs reported in the follow-up survey were significantly higher than those of the participants in the NLS group. It is also important to take the daily step counts into consideration. The significant estimate is not stronger in Model 4 than in Model 3, suggesting that our results are not due to confounding effects from daily step counts except for the side-step test.

We did not find any relationship between the vertical jump test (power) or the step-test (endurance) and the risk of LS. Although the vertical jump test also shares characteristics in common with lower extremity strength, the vertical jump test does not only measure lower extremity strength, it also depends on speed (instantaneous power) [32, 33]. The step-test assesses the ability to perform a specific muscular action for a prolonged period of time, and not just a bout of lower extremity strength [33]. Meanwhile, LS is identified as a condition in which mobility functions, such as sit-to-stand or gait, are reduced as a result of locomotive organ/system impairment [1]. It is noteworthy that the Loco-check questionnaire did not comprise items of instantaneous power and endurance; thus, the vertical jump test and the step-test might not be factors predictive of the risk of LS.

In this study, back muscle strength and grip strength did not show any association with the risk of LS. The most plausible reason for this finding was that seven questions on the Loco-check questionnaire evaluated the lower-extremity physical function status in middle-aged and older adults, and there was no question related to back muscle strength or grip strength, therefore back muscle strength and grip strength were not associated with the risk of LS.

Conversely, there was no significant relationship found between trunk lift (flexibility), standing trunk flexion (flexibility), and the risk of LS. Consistent with our results, no significant relationship between functional reach (flexibility) [34] and the risk of LS has been reported in Japanese individuals aged 40–91 years [12]. This result suggests that there is no association between flexibility and the risk of LS.

Physical fitness is defined as the ability to carry out daily tasks with vigor and alertness without undue fatigue and with ample energy to enjoy leisure-time pursuits and respond to emergencies [35]. Moreover, basic physical fitness elements include muscle strength, and muscular and circulatory endurance. Muscle power, agility, speed, and flexibility contribute to motor ability; thus, kinesthetic arm-eye foot-eye coordination is needed for general motor ability [26]. Thus, we may consider physical fitness to reflect ADL, and that motor ability is higher than physical function. In this study, none of the motor ability measurement items were associated with the risk of LS. Although the reason for these results is unclear: the seven questions on the Loco-check questionnaire designed to evaluate ADL and motor ability may not have been able to directly evaluate the ADL.

Our study has several limitations. Firstly, the current findings were not representative of all Japanese men because the study population was predominantly composed of middle-aged and older men from a single department in one university, and almost all were former university athletes. Secondly, only male alumni were included in this study. Therefore, the relationship between physical fitness, motor ability at a young age and LS risk of middle-aged and older women was not addressed. Thirdly, a self-selection bias was possible because the medical background and LS-risk test was examined using a self-administered questionnaire. In addition, since there was no information on when participants experienced the difficulty in LS, in this study, the time of the follow-up survey was regarded as the time at which they experienced the difficulty in LS. Therefore, there is a possibility that the participants had already experienced the difficulty in LS before the time of the follow-up survey. Finally, although we considered several potential confounding factors, we did not rule out the influence of current physical fitness, motor ability, and socioeconomic status. However, we considered the influence of daily step counts. However, despite these limitations, the current findings are the first to confirm the influence of physical fitness and motor ability at a young age on the progression of LS risk over a long follow-up period.

## Conclusions

Good agility (side-step test) at a young age may reduce the risk of LS in older age. Given our results, we believe that good agility at a young age is necessary to inhibit the progression of LS. In addition, our results present important clinical implications and should be taken into consideration when developing LS prevention exercise programs for young people.

## Supplementary Information

**Additional file 1.** Description of the instructions provided to participants for the execution of physical fitness tests and motor ability tests and instructions on how results were to be recorded.

**Additional file 2: Supplementary Table 1.** Physical fitness test scoring table for men. **Supplementary Table 2.** Motor ability test scoring table for men. **Supplementary Table 3.** Subjects in the present study, subjects in their fourth year at Juntendo University in 1981, and age peers in general in 1981, on physical fitness tests and motor ability tests for men. **Supplementary Table 4.** The positive rate of each question in the Loco-check.

## Data Availability

The datasets used and/or analyzed during the current study are available from the corresponding author on reasonable request.

## Abbreviations

ADL: activities of daily living
ANCOVA: analysis of covariance
BMI: body mass index
CI: Confidence interval
HR: hazard ratios
JOA: Japanese Orthopaedic Association
LS: locomotive syndrome
NLS: no locomotive syndrome
