# Supplementary material for: Association of physical fitness and motor ability at young age with locomotive syndrome risk in middle-aged and older men: J-Fit+ Study
Source: BMC Geriatr. 2021 Jan 30;21:89. doi: 10.1186/s12877-021-02047-7 (PMC7847559; doi:10.1186/s12877-021-02047-7)
Supplement: Supplementary file 1 — Additional file 1. Description of the instructions provided to participants for the execution of physical fitness tests and motor ability tests and instructions on how results were to be recorded. [file 12877_2021_2047_MOESM1_ESM.docx]

**Additional file 1**

Below is a description of the instructions provided to participants for the execution of physical fitness tests and motor ability tests and instructions on how results were to be recorded.

**Physical Fitness Tests** [1, 2]

*Side-step test (Agility)*

Stand astride over the middle line of three straight lines 120-cm apart, and execute quick stepping to the right and left sides (not jumps) for 20 seconds. Count one point each time the subject steps over each of three lines, and record the best score of two trials.

*Vertical-jump (Power)*

Draw a straight line parallel to the wall 20 cm from the wall. Stand sideways at a distance of 20 cm to the wall with the finger-tips dipped in chalk-powder, with one foot placed outside the line and the other close to the wall, mark the highest possible spot on the wall, then jump and touch the highest possible spot using fingers without approaching a run. Measure the distance between these two marks, and record the best of two trials.

*Back muscle strength (Strength)*

Stand on back with muscle strength dynamometer on the floor with feet about 15-cm apart. Hold the handle of the back measurement device with knees extended in a standing posture with 30 degrees lumbar flexion. Measure the back muscle strength by pulling the dynamometer, and record the better of two trials.

*Grip-strength (**Strength)*

Measure the grip-strength of both hands by the well adjusted and correct grip-dynamometer with the individual in the standing position. The individuals were told to keep the grip-dynamometer away from any part of the body, and encouraged to exert maximal grip effort. Measure and record the better of two trials for each hand.

*Trunk lift (Flexibility)*

Distance from floor to chin when bending the upper body upward in a face down posture. The test was completed twice and the best record was adopted.

*Trunk-forward flexion (Flexibility)*

Stand over a bench on which a stick-measure is attached, bend the trunk forward keeping both knees straight, and record the distance where both fingertips reach down as far as possible without bouncing. Performance was scored as the distance (cm) reached by the middle fingers. The upward distance from the zero mark at the level of the upper surface of the bench was a negative score and the downward distance was a positive score. Record the better distances of two trials.

*Step-test (**Endurance)*

Step up-and-down using a 40-cm high stand as an ergometer for 3 minutes. Measure the pulse three times after cessation of stepping, (a) 1–1.5 min, (b) 2–2.5 min, (c) 3–3.5 min, and record the index derived using the formula = [(Duration of stepping in second: 180 s)÷{(a+b+c)×2}]×100. Complete the test once.

**Motor Ability Tests** [1, 2]

*50-meter run*

50-meter run using a straight running track according to regular track-and-field rules. Record the time needed. Complete the test once.

*1500-meter run*

1500-meter run according to regular track-and-field rules. Record the time needed. Complete the test once.

*Running long jump*

Distance of a long jump using the sandbox with a runway. Measure the distance from the take-off point. Complete twice and record the best distance.

*Hand-ball throw*

Draw a circle of 2-m diameter, and a delta figure with 30 degree angle cross-sectioned by the circular lines 1 m apart, throw the ball (54–56 cm circumference, 325–400g weight) as far as possible within the delta area. Record the better of two trials in meters.

*Pull-up*

Pull up and flex arms on high horizontal bar approximately once every 3–4 seconds until the jaw reaches the bar, then extend the arms down, repeat the same again. Record the number of complete executions.

**REFERENCES**

1. Noi S, Masaki T. The educational experiments of school health promotion for the youth in Japan: Analysis of the 'sport test' over the past 34 years. Health Promot Int. 2002 Jun;17(2):147-160.

2. Matsushima S, Ishiko T, Matsuda I. Sports Test (in Japanese). Tokyo: DAI-ICHI HOKI; 1963.
