# Supplementary material for: Association of physical fitness and motor ability at young age with locomotive syndrome risk in middle-aged and older men: J-Fit+ Study
Source: BMC Geriatr. 2021 Jan 30;21:89. doi: 10.1186/s12877-021-02047-7 (PMC7847559; doi:10.1186/s12877-021-02047-7)
Supplement: Supplementary file 2 — Additional file 2: Supplementary Table 1. Physical fitness test scoring table for men. Supplementary Table 2. Motor ability test scoring table for men. Supplementary Table 3. Subjects in the present study, subjects in their fourth year at Juntendo University in 1981, and age peers in general in 1981, on physical fitness tests and motor ability tests for men. Supplementary Table 4. The positive rate of each question in the Loco-check. [file 12877_2021_2047_MOESM2_ESM.docx]

**Additional file 2**

**Supplementary Tables**

**Supplementary Table 1** Physical fitness test scoring table for men

| Score, point | One | Two | Three | Four | Five |
| --- | --- | --- | --- | --- | --- |
| Test items |  |  |  |  |  |
| Side-step test, point | ~31 | 32~35 | 36~41 | 42~46 | 47~ |
| Vertical jump test, cm | ~32 | 33~42 | 43~53 | 54~63 | 64~ |
| Back muscle strength, kg | ~71 | 72~107 | 108~143 | 144~177 | 178~ |
| Grip strength, kg | ~23 | 24~34 | 35~43 | 44~54 | 55~ |
| Trunk lift, cm | ~36 | 37~46 | 47~56 | 57~66 | 67~ |
| Standing trunk flexion, cm | ~4 | 5~11 | 12~18 | 19~24 | 25~ |
| Step-test^a^ | ~41.8 | 41.9~56.5 | 56.6~71.3 | 71.4~85.9 | 86.0~ |

^a^ Step-test is scored by the index derived from the formula shown in the Additional file 1.

**Supplementary Table 2** Motor ability test scoring table for men

| Test items | 50-m run, s | 1,500-m run, s | Running long jump, cm | Hand-ball throw, m | Pull-up, point |
| --- | --- | --- | --- | --- | --- |
| Score, point |  |  |  |  |  |
| Twenty | ~6.4 | ~300 | 600~ | 40 | 21~ |
| Nineteen | ~6.5 | 301~305 | 590~599 | 39 | 20 |
| Eighteen | ~6.6 | 306~310 | 580~589 | 38 | 19 |
| Seventeen | ~6.7 | 311~315 | 570~579 | 37 | 18 |
| Sixteen | ~6.8 | 316~320 | 560~569 | 36 | 17 |
| Fifteen | ~6.9 | 321~325 | 550~559 | 35 | 16 |
| Fourteen | ~7.0 | 326~330 | 540~549 | 34 | 15 |
| Thirteen | ~7.1 | 331~335 | 530~539 | 33 | 14 |
| Twelve | ~7.2 | 336~340 | 520~529 | 32 | 13 |
| Eleven | ~7.3 | 341~345 | 510~519 | 31 | 12 |
| Ten | ~7.4 | 346~350 | 500~509 | 30 | 11 |
| Nine | ~7.5 | 351~355 | 480~499 | 29 | 10 |
| Eight | ~7.6 | 356~360 | 460~479 | 28 | 9 |
| Seven | 7.7~7.8 | 361~370 | 440~459 | 27 | 8 |
| Six | 7.9~8.0 | 371~380 | 420~439 | 26 | 7 |
| Five | 8.1~8.2 | 381~390 | 400~419 | 25 | 6 |
| Four | 8.3~8.4 | 391~400 | 380~399 | 23~24 | 5 |
| Three | 8.5~8.6 | 401~410 | 360~379 | 21~22 | 4 |
| Two | 8.7~8.8 | 411~420 | 340~359 | 19~20 | 3 |
| One | 8.9~9.0 | 421~430 | 320~339 | 17~18 | 2 |

**Supplementary Table 3** Subjects in the present study, subjects in their fourth year at Juntendo University in 1981, and age peers in general in 1981, on physical fitness tests and motor ability tests for men

|  | Subjects in the present study | | |  | Subjects in their fourth year at Juntendo University in 1981 [1] | | |  | Age peers in general in 1981 [2] | | |
| --- | --- | --- | --- | --- | --- | --- | --- | --- | --- | --- | --- |
|  | n | Mean | SD |  | n | Mean | SD |  | n | Mean | SD |
| Physical fitness tests |  |  |  |  |  |  |  |  |  |  |  |
| Side-step test, point | 205 | 51.09 | (4.2) |  | 123 | 51.80 | (3.8) |  | 337 | 44.80 | (5.2) |
| Vertical jump test, cm | 205 | 63.64 | (6.8) |  | 123 | 65.90 | (6.7) |  | 337 | 58.49 | (8.3) |
| Back muscle strength, kg | 205 | 171.28 | (30.2) |  | 123 | 171.40 | (30.5) |  | 337 | 144.62 | (26.9) |
| Grip strength, kg | 205 | 51.36 | (6.4) |  | 123 | 50.90 | (5.7) |  | 335 | 49.17 | (6.3) |
| Trunk lift, cm | 205 | 58.86 | (6.8) |  | 123 | 58.30 | (5.5) |  | 249 | 55.35 | (7.7) |
| Trunk-forward flexion, cm | 205 | 14.61 | (5.7) |  | 123 | 15.90 | (4.8) |  | 339 | 13.88 | (5.6) |
| Step-test, s/s | 205 | 73.29 | (14.5) |  | 123 | 76.70 | (14.9) |  | 233 | 62.55 | (11.0) |
| Physical fitness scores, point | 205 | 28.04 | (2.1) |  | 123 | 28.50 | (2.2) |  | 231 | 25.03 | (3.2) |
| Motor ability tests |  |  |  |  |  |  |  |  |  |  |  |
| 50-m run, s | 205 | 7.08 | (0.3) |  | 123 | 7.10 | (0.4) |  | 243 | 7.25 | (0.5) |
| 1,500-m run, s | 205 | 331.62 | (31.3) |  | 123 | 324.60 | (25.6) |  | 233 | 355.82 | (32.9) |
| Running long jump, cm | 205 | 529.14 | (42.2) |  | 123 | 532.10 | (44.8) |  | 243 | 476.92 | (51.0) |
| Hand-ball throw, m | 205 | 31.48 | (4.0) |  | 123 | 33.00 | (3.9) |  | 243 | 30.46 | (4.8) |
| Pull-up, point | 205 | 14.36 | (5.4) |  | 123 | 13.10 | (5.6) |  | 241 | 10.40 | (4.3) |
| Motor ability scores, point | 205 | 64.00 | (12.1) |  | 123 | 64.90 | (12.7) |  | 232 | 50.09 | (14.8) |

The data are presented as the mean value (SD).

SD, standard deviation.

Subjects in the present study: participants who were 21 years old in the fourth year of university.

Subjects in their fourth year at university in 1981: physical fitness tests and motor ability tests of male students in the fourth year at Juntendo University in 1981.

Age peers in general in 1981: participants who were 21 years old in national physical fitness tests and motor ability tests in 1981.

REFERENCES

1. Narashino Campus Research Committee. Cumulative Record on Physique, Physical Fitness and Motor Ability of Male Students of Juntendo University Narashino Campus (in Japanese). Juntendo University Bulletin of Health and Physical Education. 1981 Dec; 24:108-109.

2. Ministry of Education, Physical Education Bureau. The Report of FY1981 Survey on Physical Strength and Athletic Performance (in Japanese). Tokyo: Author; 1982.

**Supplementary Table 4** The positive rate of each question in the Loco-check

| Statements in the Loco-check questionnaire | n (%) |
| --- | --- |
| 1. You cannot put on your sock standing on one leg | 10 (4.3%) |
| 2. You often trip or slip around the house | 10 (4.3%) |
| 3. You need to hold on to the handrail when climbing the stairs | 12 (5.2%) |
| 4. You have difficulty doing moderately heavy housework | 4 (1.7%) |
| 5. You have difficulty carrying home 2 kg of shopping  (e.g., equivalent to two 1-L cartons of milk) | 4 (1.7%) |
| 6. You cannot walk for a quarter of an hour nonstop | 3 (1.3%) |
| 7. You cannot make it across the road before the light turns red | 15 (6.5%) |

The data are presented as number (percentage).
